# Long-Lasting Impairments in Quadriceps Mitochondrial Health, Muscle Size, and Phenotypic Composition Are Present After Non-invasive Anterior Cruciate Ligament Injury

**DOI:** 10.3389/fphys.2022.805213

**Published:** 2022-01-28

**Authors:** Steven M. Davi, Ahram Ahn, McKenzie S. White, Timothy A. Butterfield, Kate Kosmac, Oh Sung Kwon, Lindsey K. Lepley

**Affiliations:** ^1^ Department of Kinesiology, University of Connecticut, Storrs, CT, United States; ^2^Department of Orthopedic Surgery, John A. Feagin Jr Sports Medicine Fellowship, Keller Army Hospital, West Point, NY, United States; ^3^School of Kinesiology, University of Michigan, Ann Arbor, MI, United States; ^4^Center for Muscle Biology, University of Kentucky, Lexington, KY, United States; ^5^Department of Athletic Training and Clinical Nutrition, University of Kentucky, Lexington, KY, United States; ^6^ Department of Physical Therapy, University of Kentucky, Lexington, KY, United States; ^7^Department of Orthopaedic Surgery and Center on Aging, University of Connecticut School of Medicine, Farmington, CT, United States

**Keywords:** ACL, redox disturbance(s), muscle atrophy, quadriceps, mitochondria

## Abstract

**Introduction:**

Despite rigorous rehabilitation aimed at restoring muscle health, anterior cruciate ligament (ACL) injury is often hallmarked by significant long-term quadriceps muscle weakness. Derangements in mitochondrial function are a common feature of various atrophying conditions, yet it is unclear to what extent mitochondria are involved in the detrimental sequela of quadriceps dysfunction after ACL injury. Using a preclinical, non-invasive ACL injury rodent model, our objective was to explore the direct effect of an isolated ACL injury on mitochondrial function, muscle atrophy, and muscle phenotypic transitions.

**Methods:**

A total of 40 male and female, Long Evans rats (16-week-old) were exposed to non-invasive ACL injury, while 8 additional rats served as controls. Rats were euthanized at 3, 7, 14, 28, and 56 days after ACL injury, and vastus lateralis muscles were extracted to measure the mitochondrial respiratory control ratio (RCR; state 3 respiration/state 4 respiration), mitochondrial reactive oxygen species (ROS) production, fiber cross sectional area (CSA), and fiber phenotyping. Alterations in mitochondrial function and ROS production were detected using two-way (sex:group) analyses of variance. To determine if mitochondrial characteristics were related to fiber atrophy, individual linear mixed effect models were run by sex.

**Results:**

Mitochondria-derived ROS increased from days 7 to 56 after ACL injury (30–100%, *P* < 0.05), concomitant with a twofold reduction in RCR (*P* < 0.05). Post-injury, male rats displayed decreases in fiber CSA (days 7, 14, 56; *P* < 0.05), loss of IIa fibers (day 7; *P* < 0.05), and an increase in IIb fibers (day 7; *P* < 0.05), while females displayed no changes in CSA or phenotyping (*P* > 0.05). Males displayed a positive relationship between state 3 respiration and CSA at days 14 and 56 (*P* < 0.05), while females only displayed a similar trend at day 14 (*P* = 0.05).

**Conclusion:**

Long-lasting impairments in quadriceps mitochondrial health are present after ACL injury and play a key role in the dysregulation of quadriceps muscle size and composition. Our preclinical data indicate that using mitoprotective therapies may be a potential therapeutic strategy to mitigate alterations in muscle size and characteristic after ACL injury.

## Introduction

Despite rigorous rehabilitation aimed at restoring muscle health, anterior cruciate ligament (ACL) injury is often hallmarked by significant long-term muscle weakness (Lepley, 2015; Otzel et al., 2015; Cinque et al., 2017). It is well known that the strength deficits that occur immediately following ACL injury result from a complex cascade of neurological (Ingersoll et al., 2008; Pietrosimone et al., 2012) and inflammatory events (Jackman and Kandarian, 2004; Mendias et al., 2013; Peck et al., 2019; Hunt et al., 2020) that negatively influence muscle health. Long-term alterations in quadriceps muscle volume (Kuenze et al., 2016; Norte et al., 2018; Lepley et al., 2019), fiber area (Noehren et al., 2016; Gumucio et al., 2018), and fibrotic tissue content (Noehren et al., 2016; Peck et al., 2019) have also been documented. Despite the continually increasing knowledge base, therapeutic strategies that effectively treat muscle weakness remain elusive (Lepley, 2015; Otzel et al., 2015). Further identification of the mechanistic factors that compromise muscle health is critical for the advancement of evidence-based therapies to counteract the significant muscle weakness that plagues many of the 300,000 patients who experience an ACL injury each year (Griffin et al., 2006).

Mitochondria are critical organelles responsible for regulating many critical cellular processes in skeletal muscle. For instance, mitochondria play central roles in muscle metabolism (Osellame et al., 2012; Seo et al., 2016), regulation of energy supply and signaling (Powers et al., 2012; Romanello and Sandri, 2013), reactive oxygen species (ROS) production (Davies et al., 1982; Koren et al., 1983), calcium homeostasis (Powers et al., 2011), and apoptosis (Sandri, 2008; Romanello and Sandri, 2013). Consequently, it is not surprising that derangements in their function is a common feature of many atrophying conditions (e.g., bed rest, disuse, and aging) (Powers et al., 2012; Talbert et al., 2013; Tryon et al., 2014; Park et al., 2018). Yet, the extent to which mitochondria are involved in the detrimental sequela of quadriceps muscle after ACL injury is not well understood. Early data indicate a loss of mitochondria density within the quadriceps after ACL reconstruction (Fluck et al., 2018), which may impair oxygen uptake, and utilization, leading to energy supply deficits. Though insightful (Fluck et al., 2018), further efforts are needed to solidify the role of muscle mitochondria after ACL rupture. Of particular importance is studying adaptations in quadriceps mitochondrial respiratory capacity, mitochondria damage, and the expression of mitochondrial ROS after injury, as these events all are implicated in muscle atrophy (Powers et al., 2012; Romanello and Sandri, 2013) and can be targeted with mitoprotective treatments. Muscle phenotypic transitions that occur after ACL injury may also be closely linked with mitochondrial health (Babcock et al., 2015; Noehren et al., 2016).

On the basis of the culminating evidence (Osellame et al., 2012; Fluck et al., 2018), and the strong physiological link between mitochondrial health and adaptations in muscle size and composition, it stands that mitochondria may play a key role in the dysregulation of skeletal muscle health after ACL injury. Thus, the purpose of this study was to explore the direct effect of an isolated ACL injury on mitochondrial function, mitochondria-derived ROS, muscle atrophy, and muscle phenotyping, utilizing a pre-clinical non-invasive rodent ACL injury model. Like other musculoskeletal pathologies tied to mitochondrial dysfunction, we hypothesized mitochondrial respiratory function would worsen over time after ACL injury concomitantly with increased mitochondrial derived ROS production. Further, we anticipate that maladaptive mitochondrial function would be related to changes in muscle fiber size and the phenotypic characteristics.

## Materials and Methods

### Animals and Non-invasive Anterior Cruciate Ligament Injury

A total of 48 Long Evans rats (24 male, 24 females; 16 weeks) were obtained (Envigo Laboratories, Indianapolis, IN, United States) under the approval of the Institutional Animal Care and Use Committee (IACUC approval #A17-042) at the University of Connecticut. Animals were maintained in accordance with the National Institutes of Health guidelines on the care and use of laboratory animals. All animals were housed in standard sized cages on a 12:12 light–dark cycle and allowed both food and water *ad libitum* for the duration of the study. An *a priori* power analysis was conducted based on previous animal (Jones et al., 2010; Haslauer et al., 2014; Bigoni et al., 2016; Lepley et al., 2016; Wurtzel et al., 2017) and human studies (Mendias et al., 2013; Lepley et al., 2015) that explored the influence of a variety of factors on muscle size and composition after ACL injury. A sample size of 6 rats per groups was determined to be sufficient to detect large effect sizes (*d* ≤ 0.8, *P* = 0.05) and an additional 2 rats per group were included to account for potential attrition.

A total of 40, 16-week-old Long Evans rats were exposed to non-invasive rupture of the ACL that was induced by tibial compression overload. Animals were euthanized and tissues collected at 3, 7, 14, 28, 56 days following injury (White et al., 2020). ACL rupture was confirmed upon post-mortem dissection by two trained examiners (SD and LL). Eight rats served as healthy controls (HC, aged-matched to the 56 day group). The right vastus lateralis (VL) of the ACL-injured hindlimb was dissected immediately following euthanasia (*via* carbon dioxide overdose) and a small section was taken from the distal portion of the muscle to be used for measurement of mitochondrial respiration (∼25 μg), while the remaining muscle was flash frozen in liquid nitrogen and stored at −80°C for future immunohistochemical assays.

### Mitochondrial Respiration

Fresh tissue samples were first immersed in preservation fluid [BIOPS; containing (in mM) 2.77 CaK2EGTA, 7.23 K2EGTA, 6.56 MgCl_2_, 0.5 DTT, 50 K-MES, 20 imidazole, 20 taurine, 5.77 Na2ATP, and 15 phosphocreatine, pH 7.1 at 4°C] for a minimum of 30 min (Kuznetsov et al., 2008). Fresh fiber bundles were gently separated along their longitudinal axis on ice in cold BIOPS [containing (in mM) 2.77 CaK2EGTA, 7.23 K2EGTA, 6.56 MgCl_2_, 0.5 DTT, 50 K-MES, 20 imidazole, 20 taurine, 5.77 Na2ATP, and 15 phosphocreatine, pH 7.1] using needle-tipped forceps under magnification (Laxco, Mill Creek, WA, United States). Permeabilized fibers were then incubated in BIPOS buffer containing 50 μg/ml saponin for 30 min at 4°C. Following saponin treatment, samples were washed for 5 min three times in MIR05 [containing (in mM) 2.77 CaK2EGTA, 7.23 K2EGTA, 6.56 MgCl_2_, 0.5 DTT, 20 imidazole, 5.77 ATP, 15 phosphocreatine, 50 K-MES, and 20 taurine, pH 7.0] in preparation for high-resolution respirometry.

Mitochondrial respiratory function was assessed using a high-resolution respirometry (Oroboros O2k, Innsbruck, Austria) with a protocol by Park et al. (2018). Briefly, after a 10 min period of equilibration (e.g., tissue immersed in 2 ml of MIR05 while being continuously stirred at 37°C) O_2_ consumption (pmol s^–1^) of duplicate samples was continuously recorded as mitochondria complex agonist and antagonist substrates (2 mM glutamate–10 mM malate, 5 mM ADP, 10 mM succinate, 0.5 μM rotenone, 10 μM cytochrome c, and 2 g/ml oligomycin) were exposed to each sample. Complex I + II (CI + II) state 3 respiration (glutamate, malate, ADP, and succinate) and state 4 respiration (blocking of ATP synthase *via* oligomycin) were assessed. The respiratory control ratio (RCR) was calculated by dividing state 3 by state 4 respiration (Park et al., 2018).

### Mitochondrial ROS Production

ROS emission in permeabilized muscle fibers was determined using Amplex Red (Molecular Probes, Eugene, OR, United States). This assay is based on the concept that in permeabilized muscle fibers horseradish peroxidase catalyzes the H_2_O_2_-dependent oxidation of non-fluorescent Amplex Red to fluorescent resorufin red (Anderson and Neufer, 2006; Anderson et al., 2009). Superoxide dismutase was added to the preparation to convert all superoxide into H_2_O_2_. Fiber bundles were then incubated at 37°C in 96-well plates in reaction buffer (5 mM succinate, 10 mM MgCl_2_, 10 mM KH_2_PO_4_, 100 mM KCl, 50 mM MOPS, 1 mM EGTA, 0.4 μg/ml BSA, 0.02 U horseradish peroxidase, 2.5 μg Amplex Red, pH 7.0) (Min et al., 2015; Talbert et al., 2016). After 30 min of incubation, the amount of fluorescent resorufin produced was measured using an excitation wavelength of 545 nm and an emission wavelength of 590 nm in flurometric multiwell-plate reader (SpectraMax, Molecular Devices, Sunnyvale, CA, United States). Mitochondrial ROS production was measured during state 3 respiration using the creatine kinase energy clamp technique to maintain respiration at a steady state (Messer et al., 2004).

### Muscle Phenotyping

Frozen mid-belly cross-sections of the VL were sectioned (6 μm) and mounted on glass slides. Slides were then prepped [e.g., air-dried overnight and then washed in phosphate buffered saline (PBS) 3 × 5 min] and immunoreacted in a primary antibody cocktail for muscle fiber type expression [Type I: BA.D5-C IgG2b (1:100), DHSB University of Iowa, Iowa City, IA, United States; Type IIa: SC.71 IgG1 (1:2), DHSB University of Iowa, Iowa City, IA, United States; Type IIb: BF.F3 IgM (1:2), DHSB University of Iowa, Iowa City, IA, United States; Laminin, Sigma #L9393 (1:50), Sigma-Aldrich, St. Louis, MO, United States] overnight at 4°C. Slides were then washed (three times in PBS solution) before the application of an Laminin amplifier [Biotin-SP AffiniPure Goat Anti-Rabbit IgG (1:500), Jackson Laboratories, West Grove, PA, United States] for 90 min at room temperature. After Laminin amplification, to visualize fiber fluorescent expression, slides were incubated in a secondary antibody cocktail diluted in PBS [Type I: Gt anti-Ms IgG2b, Alexa Fluor 647 (1:250) Type IIa: Gt anti-Ms IgG1, Alexa Fluor 488 (1:500); Type IIb: Gt anti-Ms IgM, Alexa Fluor 555 (1:250), Invitrogen; Laminin: Streptavidin-AMCA (1:250), Vector Laboratories, Burlingame, CA, United States] for 1 h at room temperature. Following incubation, slides were fixed in mounting medium (H-1000, Vector Laboratories, Burlingame, CA, United States) and cover-slipped. Imaging was subsequently captured at 100× on five random fields of view (LSM 880, Carl Zeiss, Göttingen, Germany) and analyzed with ZEN blue software (Carl Zeiss, Göttingen, Germany). Fiber type distribution and fiber cross sectional area (CSA) were assessed with MyoVision (University of Kentucky, Lexington, KY, United States) (Wen et al., 2018).

### Statistical Analyses

First, to determine if respiratory function and mitochondrial ROS production worsens over time after ACL injury, two-way analyses of variance were used to evaluate the effect of time from ACL injury and sex on RCR, state 3 and state 4 respiration, and ROS emission values. When appropriate, significant interactions were further evaluated using separate one-way ANOVAs with Dunnett *post hoc* tests relative to sex-matched controls. Next, to characterize the degree of atrophy and phenotypic alterations after ACL injury, histograms representing the fiber type frequency as a function of CSA were created. Only fiber types with >5% overall dispersion were included in subsequent analyses. Average fiber type CSA and fiber type percent distributions were also compared between ACL-injured and controls rats by sex with independent *t*-tests. Finally, to determine if maladaptive mitochondrial characteristics were related to reductions in fiber size, individual linear mixed effect models with a random effect of rat were used to investigate the influence of mitochondrial function on muscle size after ACL injury in males and females. Significance was set *a priori* at an alpha level of *P* < 0.05 and all analyses were performed using RStudio (version 1.4.7).

## Results

**FIGURE 1:**
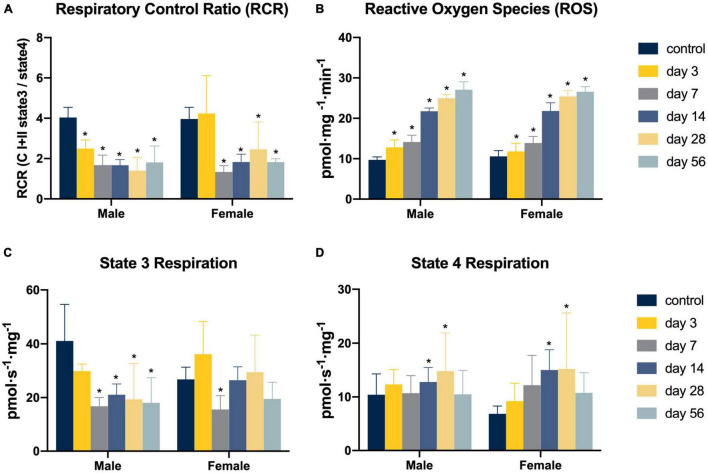
Mitochondrial function following non-invasive ACL injury. **(A–D)** Mitochondria respiratory function and ROS production. Eight Long Evans rats (4m/4f) were allocated to each group. Two-way analyses of variance (ANOVA) and *post hoc* one-way ANOVAs with Dunnett tests were used to evaluate the effect of time from ACL injury and sex on RCR, state 3 and state 4 respiration, and ROS emission values. Asterisk denotes significant difference from control group (*P* < 0.05).

Overall, a longitudinal reduction in mitochondrial RCR was found to be present in conjunction with a longitudinal increase in ROS production after ACL injury (see Figure 1). Specifically compared to controls, ACL injured males in the days 7–56 injury groups demonstrated reductions in RCR (control: 4.04 ± 0.42; day 3: 2.50 ± 0.32, mean difference = −1.54, *P* = 0.01, 95% CI: −2.74, −0.35, *d* = 4.12; day 7: 1.74 ± 0.53, mean difference = −2.30, *P* < 0.01, 95% CI: −3.40, −1.19, *d* = 4.18; day 14: 1.67 ± 0.23, mean difference = −2.37, *P* < 0.01, 95% CI: −3.48, −1.26, *d* = 7.00; day 28: 1.40 ± 0.70, mean difference = −2.64, *P* < 0.01, 95% CI: −3.74, −1.53, *d* = 4.57; day 56: 1.81 ± 0.85, mean difference = −2.23, *P* < 0.01, 95% CI: −3.33, −1.12, *d* = 3.33) which appeared to be primarily driven by lower state 3 respiration (control: 41.09 ± 13.80 pmol s^–1^ mg^–1^; day 7: 16.32 ± 3.47 pmol s^–1^ mg^–1^, mean difference = −24.76, *P* < 0.01, 95% CI: −41.99, −7.53, *d* = 2.46; day 14: 21.07 ± 3.37 pmol s^–1^ mg^–1^, mean difference = −20.02, *P* = 0.02, 95% CI: −37.25, −2.79, *d* = 1.99; day 28: 19.38 ± 11.97 pmol s^–1^ mg^–1^, mean difference = −21.71, *P* = 0.01, 95% CI: −38.93, −4.48, *d* = 1.68; day 56: 18.07 ± 8.67 pmol s^–1^ mg^–1^, mean difference = −23.02, *P* < 0.01, 95% CI: −40.25, −5.79, *d* = 2.00). ACL injured females in the days 7–56 injury groups demonstrated reductions in RCR (control: 3.97 ± 0.36; day 7: 1.34 ± 0.32, mean difference = −2.63, *P* < 0.01, 95% CI: −3.96, −1.30, *d* = 7.72; day 14: 1.83 ± 0.41, mean difference = −2.14, *P* < 0.01, 95% CI: −3.46, −0.81, *d* = 5.55; day 28: 2.46 ± 0.63, mean difference = −1.51, *P* = 0.02, 95% CI: −2.83, −0.18, *d* = 2.94; day 56: 1.83 ± 0.15, mean difference = −2.14, *P* < 0.01, 95% CI: −3.47, −0.81, *d* = 7.76) also likely driven by lower state 3 respiration (control: 26.75 ± 3.33 pmol s^–1^ mg^–1^; day 7: 15.58 ± 4.81 pmol s^–1^ mg^–1^, mean difference = −11.17, *P* = 0.03, 95% CI: −21.41, −0.93, *d* = 2.70). No difference in state 4 respiration was detected (*P* > 0.05). Finally, ROS production was significantly elevated compared to controls in all ACL injury groups in males (control: 9.72 ± 0.74 pmol mg^–1^ min^–1^; day 3: 12.81 ± 1.84 pmol mg^–1^ min^–1^, mean difference = 3.09, *P* = 0.04, 95% CI: 0.16, 6.02, *d* = 2.20; day 7: 14.16 ± 1.67 pmol mg^–1^ min^–1^, mean difference = 4.45, *P* < 0.01 95% CI: 1.73, 7.15, *d* = 3.44; day 14: 21.75 ± 3.37 pmol mg^–1^ min^–1^, mean difference = 12.03, *P* < 0.01, 95% CI: 9.32, 14.74, *d* = 4.93; day 28: 25.00 ± 0.85 pmol mg^–1^ min^–1^, mean difference = 15.28, *P* < 0.01, 95% CI: 12.56, 17.99, *d* = 19.17; day 56: 27.08 ± 1.96 pmol mg^–1^ min^–1^, mean difference = 17.36, *P* < 0.01, 95% CI: 14.64, 20.07, *d* = 11.72) and females in days 7–56 (control: 10.59 ± 1.43 pmol mg^–1^ min^–1^; day 7: 13.89 ± 1.61 pmol mg^–1^ min^–1^, mean difference = 3.30, *P* = 0.05, 95% CI: 0.05, 6.55, *d* = 2.17; day 14: 21.81 ± 2.04 pmol mg^–1^ min^–1^, mean difference = 11.22, *P* < 0.01, 95% CI: 7.97, 14.46, *d* = 6.37; day 28: 25.42 ± 1.46 pmol mg^–1^ min^–1^, mean difference = 14.83, *P* < 0.01, 95% CI: 11.58, 18.08, *d* = 10.26; day 56: 26.59 ± 1.22 pmol mg^–1^ min^–1^, mean difference = 15.99, *P* < 0.01, 95% CI: 12.75, 19.24, *d* = 12.04).

**FIGURE 2:**
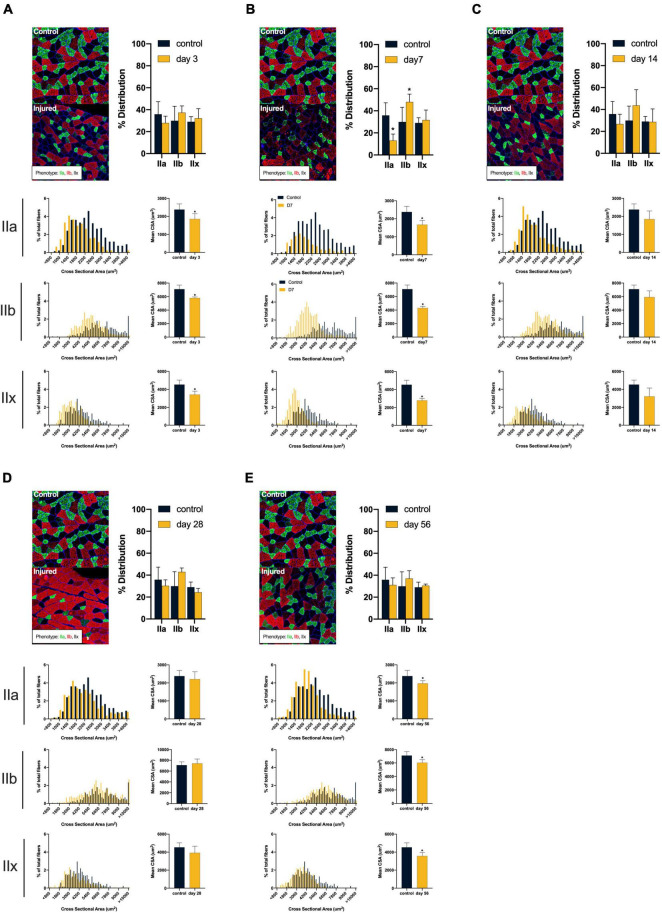
*Males*: Alterations in quadriceps composition following ACL injury. **(A–E)** Longitudinal summary of male rat muscle fiber size and phenotypic characteristics after ACL injury compared to male control. Representative images of vastus lateralis muscle stained for muscle fiber typing with percent distributions and histograms representing the fiber type frequency as a function of CSA. The healthy control group consisted of 8 rats (4m/4f) age-matched to the day 56 injury group. As such, a single representative image was selected from the male control group and is presented in comparison to all post-injury time points. Asterisk denotes significant mean difference between ACL injured group(s) and control rats (*P* < 0.05).

**FIGURE 3:**
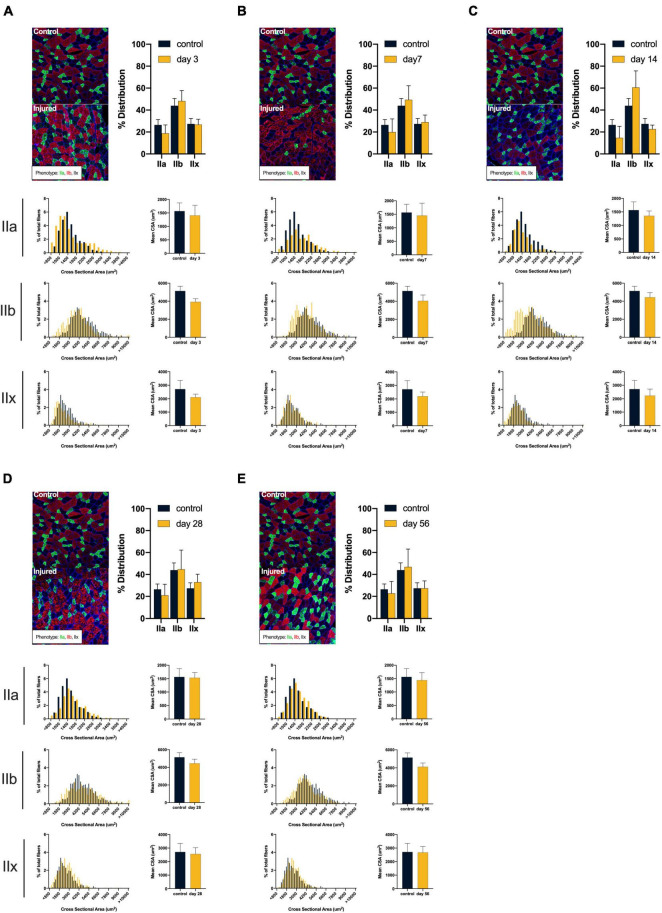
*Females*: Alterations in quadriceps composition following ACL injury. **(A–E)** Longitudinal summary of male rat muscle fiber size and phenotypic characteristics after ACL injury compared to male control. Representative images of vastus lateralis muscle stained for muscle fiber typing with percent distributions and histograms representing the fiber type frequency as a function of CSA. The healthy control group consisted of 8 rats (4m/4f) age-matched to the day 56 injury group. As such, a single representative image was selected from the female control group and is presented in comparison to all post-injury time points. No significant mean differences were detected between ACL injured group(s) and control rats (*P* < 0.05).

ACL injury was found to introduce a shift toward faster and smaller muscle fiber types after injury (see Figure 2). Compared to controls, ACL injured males at days 3, 7, and 56 displayed atrophy of type IIa (control: 2378.27 ± 313.27 μm^2^; day 3: 1847.56 ± 296.93 μm^2^, mean difference = 530.71, *P* = 0.05, 95% CI: 2.63, 1058.79, *d* = 1.74; day 7: 1672.34 ± 229.57 μm^2^, mean difference = 705.93, *P* = 0.01, 95% CI: 230.77, 1181.10, *d* = 2.57; day 56: 1969.20 ± 149.12 μm^2^, mean difference = 409.07, *P* = 0.05, 95% CI: −15.40, 833.54, *d* = 1.67), type IIb (control: 7099.53 ± 604.70 μm^2^; day 3: 5787.31 ± 155.56 μm^2^, mean difference = 1321.22, *P* < 0.01, 95% CI: 548.30, 2076.13, *d* = 2.97; day 7: 4301.06 ± 249.43 μm^2^, mean difference = 2798.47, *P* < 0.01, 95% CI: 1998.18, 3598.77, *d* = 6.05; day 56: 6035.45 ± 444.99 μm^2^, mean difference = 1064.80, *P* = 0.03, 95% CI: 145.52, 1982.63, *d* = 2.00), and type IIx (control: 4536.08 ± 498.86 μm^2^; day 3: 3416.29 ± 382.23 μm^2^, mean difference = 1119.79, *P* = 0.01, 95% CI: 350.90, 1888.69, *d* = 2.52; day 7: 2786.92 ± 222.54 μm^2^, mean difference = 1749.17, *P* < 0.01, 95% CI: 1080.86, 2417.48, *d* = 4.53; day 56: 3571.68 ± 386.50 μm^2^, mean difference = 964.41, *P* = 0.02, 95% CI: 192.33, 1736.49, *d* = 2.16) fibers. Additionally, injured males at day 7 displayed a reduction in type IIa distribution (control: 35.86 ± 11.44%; day 7: 13.11 ± 5.82%, mean difference = 22.75, *P* = 0.01, 95% CI: 7.04, 38.45, *d* = 2.51) in conjunction with an increase in type IIb distribution (control: 29.06 ± 13.21%; day 7: 48.08 ± 7.07%, mean difference = −18.15, *P* = 0.05, 95% CI: −36.47, 0.19, *d* = 1.80). Conversely, ACL injured females did not display changes in either fiber size or distribution (*P* > 0.05, see Figure 3). Both type I and hybrid fiber types were identified during image processing but failed to meet the >5% threshold and were subsequently removed analyses.

The linear mixed effect models revealed a link between mitochondrial state 3 respiration and fiber CSA after ACL injury. Specifically, males displayed a positive relationship between state 3 respiration and fiber CSA at days 14 (*P* = 0.03) and 56 (*P* = 0.02) indicating lower oxygen consumption was related to smaller fiber CSA. Females displayed a similar trend, but only at day 14 (*P* = 0.05). These observations directly support our overarching premise that mitochondrial health is key to explaining quadriceps morphology after ACL injury.

## Discussion

The concept that mitochondrial dysfunction initiates or is involved in protracted muscle dysfunction is novel to ACL injury, but has been well-established in other fields of muscular dysfunction research (Min et al., 2011; Powers et al., 2012; Stephenson and Hawley, 2014; Tryon et al., 2014; Seo et al., 2016). The present study provides clear evidence that long-lasting impairments in quadriceps mitochondrial health are present and play a key role in the dysregulation of quadriceps muscle size and composition after ACL injury. Specifically, we observed severe reductions in quadriceps mitochondrial RCR that started at day 7 after ACL injury and continued through day 56. The RCR was driven by lower state 3 respiration, implicating a lack of maximal oxygen uptake capacity. Our poor respiratory function occurred alongside the excessive mitochondrial production of ROS, as we also observed a 30–100% increased production of H_2_O_2_ expression between 7 and 56 days after injury. Muscle atrophy and alterations in fiber phenotyping were detected following ACL injury, most prominently in males. Direct links also provide evidence that the intricate balance of oxidative fiber phenotypes and CSA are impacted by reductions in mitochondrial oxygen capacity after ACL injury.

Prior cross-sectional work has identified a loss of quadriceps mitochondrial density after ACL reconstruction (Fluck et al., 2018), which can impair muscular oxygen handling. The power of our experimental design is that we were able to directly link impairments in mitochondrial function to adaptations in muscle and outline the time course of mitochondrial dysfunction following ACL injury. These data will help to establish windows of opportunity for future interventions. Our work shows that there is considerable mitochondrial dysfunction and ROS production after ACL injury that is long-lasting. These breakthrough data may be key to explaining the complex manifestation of quadriceps weakness after ACL injury, as elements of muscle health after ACL injury that have yet to be fully explained (contractile dysfunction, damage, and atrophy), are strongly associated with reductions in RCR and high ROS production (Powers et al., 2012; Stephenson and Hawley, 2014; Steinbacher and Eckl, 2015; Park et al., 2018). In our preclinical model, reductions in state 3 respiration were found to be the primary driver of impaired RCRs. This result indicates that the muscle is unable to process locally available oxygen, likely leading to widespread energy deficits, which can disrupt protein synthesis and has been linked to disuse-related muscle atrophy (Senf et al., 2010; Smuder et al., 2015). While ACL injury-induced changes in oxygen handling remain a novel concept, Jaffri et al. (2021) recently identified reductions in quadriceps oxygen consumption during exercise after ACL reconstruction, despite the availability of muscular oxygen. Although no determination was made about why quadriceps oxygen stores were underutilized, data from the preset study would suggest that one possible explanation is a loss of maximal mitochondrial oxygen consumption. In addition to reductions in oxygen consumption the steady longitudinal increase in ROS production is equally troubling, as excessive production of ROS leads to a disruption of proteostasis (protein synthesis to degradation ratios) and causes muscle contractile dysfunction (Steinbacher and Eckl, 2015). As such, mitochondrial dysfunction and redox disturbances appear to be key factors to explaining the compromised quadriceps muscle health that is commonly observed after ACL injury. Clinically, these data open the door to different mitoprotective therapeutic interventions. For instance, the use of antioxidants to mitigate the effects of ROS and promote mitochondrial health (Min et al., 2011) might be viable future therapeutic options for the field to explore.

ACL injury commonly produces a sustained loss of muscle volume, but the underlying atrophic mechanisms have yet to be thoroughly defined. The present data provides evidence that alterations in mitochondrial health are related to reductions in muscle fiber size and distribution. Specifically, type IIa fibers were found to be the most suspectable to alteration after ACL injury displaying both significant atrophy and changes in phenotypic dispersion (Picard et al., 2012). This preclinical work aligns well with the current clinical observations, as humans with ACL injury have also been shown to have selective atrophy of type IIa fibers after ACL injury and reconstruction (Noehren et al., 2016). The signaling pathways linking mitochondrial dysfunction to muscle atrophy are complex. For example, excessive release of the mitochondrial-derived proteins, apoptosis inducing factor and cytochrome-C by damaged or dysfunctional mitochondria can result in the upregulation of caspase-3 (Hyatt et al., 2019), which can increase the susceptibility of contractile protein elements to be targeted for degradation (Powers et al., 2011). Specifically, caspase-3 pathways degrade structural proteins, such as titin and nebulin, thereby releasing actin and myosin for degradation *via* the ubiquitin proteasome system (Huang and Forsberg, 1998; Du et al., 2004; Talbert et al., 2013).

The apparent disruption of type IIa fibers in the present study is particularly interesting when considering the notable loss of type IIa contractile function which has been identified by others following ACL injury (Gumucio et al., 2018; Tourville et al., 2021). Specifically, biopsied type IIa fibers displayed an independent loss of force production which was unrelated to neurologic activation (Gumucio et al., 2018; Tourville et al., 2021). The authors speculated that underlying processes of protein degradation were responsible for a decline in myofibril density, which ultimately reduced optimal contractile function. Our data may provide context to post-ACL injury contractile dysfunction as poor mitochondrial health has been related to lower energy availability for use during contraction, and generation of excessive ROS production which can instigate protein degradation processes (Huang and Forsberg, 1998; Powers et al., 2012; Talbert et al., 2013; Steinbacher and Eckl, 2015). As such, mitochondrial health may play an important role in contractile dysfunction after ACL injury and likely warrants future targeted research.

The effects of ACL injury on mitochondrial function, fiber size and phenotypic dispersion were found to vary by sex. Generally, males displayed a greater overall reduction in fiber CSA across phenotypes and a more dramatic shift in phenotype distributions. These changes were most apparent at day 7 after injury where the greatest fiber atrophy was identified and a switch in distribution toward glycolytic phenotypes occurred (see Figure 2). Interestingly, females appeared to be more resistive to atrophy and phenotypic changes despite obvious alterations in mitochondrial function. While we are unable to directly address the underlying source(s) of these sex-based differences, plausible hypotheses can be inferred. Skeletal muscle mitochondria contain receptor sites for estrogen and progesterone (Rosa-Caldwell and Greene, 2019), both of which can enhance mitochondrial metabolic efficiency and energy production (Rosa-Caldwell and Greene, 2019). Additionally, estrogen has been found to aid in muscle recovery from disuse related atrophy (McClung et al., 2006). As such, females may possess a protective hormonal mechanism to mitochondria-related atrophy.

The present study was not without limitation. First, while a statistical link was established between mitochondrial dysfunction and alterations in muscle composition, we did not evaluate how underlying pathways of muscle atrophy were affected. Oxidative capacity and ROS production have been implicated with muscle atrophy through calpain and caspase-3 pathways, which underlying triggers for accelerated protein degradation (Talbert et al., 2013). Future work should seek to identify a potential mechanism linking mitochondrial and muscular health after ACL injury. Second, our data describe non-invasive ACL injury without subsequent reconstructive surgery, which is commonly performed in humans. While there is precedent for phenotypic alteration prior to surgery (Noehren et al., 2016), the present work will need to be translated to post-surgical cases in order to better account for alterations in quadriceps mitochondrial health and muscle composition related to surgical intervention. Third, cage activity (i.e., walking) was not monitored in this cohort of rats. Our previous work in the same cohort established negative changes in movement quality (White et al., 2020), which may be related to the underlying alterations in muscle composition that were identified in the present work and requires future evaluation. Finally, mitochondrial abundance was not evaluated in the present study due to the novel exploratory nature of the work. Now that we know that mitochondrial function is impaired and associated with shifts in phenotypic composition after ACL injury, ascertaining whether the abundance of mitochondria is also altered is an important next step.

## Conclusion

The concept that mitochondrial dysfunction initiates or is involved in the detrimental sequela of muscle dysfunction is novel to ACL injury but is well-established and consistent with other areas. The present study provides clear evidence that long-lasting impairments in quadriceps mitochondrial health are present after ACL injury and likely play a key role in the dysregulation of quadriceps muscle size and composition. Since mitochondrial dysfunction plays a central role in directing muscle atrophy and is elevated after ACL injury, our preclinical data indicate that targeting mitochondria may be a potential therapeutic strategy against muscle weakness in patients with torn ACLs.

## Data Availability Statement

The raw data supporting the conclusions of this article will be made available by the authors, without undue reservation.

## Ethics Statement

The animal study was reviewed and approved by the University of Connecticut IACUC.

## Author Contributions

SD, OK, and LL designed and conceived the study, and performed all primary experimentation. AA and MW assisted carrying out data collection and analysis. TB and KK provided technical expertise and assisted in analysis for muscle phenotypic measurements. SD organized the database, performed the statistical analysis, and drafted all sections of the manuscript. LL and OK assisted in writing sections of the manuscript and providing primary revisions. MW, TB, and KK provided edits and feedback on late versions of the manuscript. All authors contributed to manuscript revision, read, and approved the submitted version.

## Conflict of Interest

The authors declare that the research was conducted in the absence of any commercial or financial relationships that could be construed as a potential conflict of interest.

## Publisher’s Note

All claims expressed in this article are solely those of the authors and do not necessarily represent those of their affiliated organizations, or those of the publisher, the editors and the reviewers. Any product that may be evaluated in this article, or claim that may be made by its manufacturer, is not guaranteed or endorsed by the publisher.
